# Electrospun Polyimide Nanofibers Modified with Metal Oxide Nanowires and MXene for Photocatalytic Water Purification

**DOI:** 10.3390/nano15171371

**Published:** 2025-09-05

**Authors:** Andrii Lys, Valerii Myndrul, Mykola Pavlenko, Błażej Anastaziak, Pavel Holec, Kateřina Vodseďálková, Emerson Coy, Mikhael Bechelany, Igor Iatsunskyi

**Affiliations:** 1 NanoBioMedical Centre, Adam Mickiewicz University, 61-712 Poznan, Poland; andrii.lys@amu.edu.pl (A.L.); mykpav@amu.edu.pl (M.P.); blazej.anastaziak@amu.edu.pl (B.A.); coyeme@amu.edu.pl (E.C.); 2 Sensor Engineering Department, Faculty of Science and Engineering, Maastricht University, P.O. Box 616, 6200 MD Maastricht, The Netherlands; valerii.myndrul@maastrichtuniversity.nl; 3 Department of Nonwovens and Nanofibrous Materials, Faculty of Textile Engineering, Technical University of Liberec, 461 17 Liberec, Czech Republic; pavel.holec@tul.cz; 4 Nanopharma a.s., 530 09 Pardubice, Czech Republic; vodsedalkova@nanopharma.cz; 5 European Institut of Membranes (IEM)—UMR 5635, University of Montpellier, ENSCM, CNRS, 34090 Montpellier, France; mikhael.bechelany@umontpellier.fr

**Keywords:** electrospinning, nanofibers, MXene (Ti_3_C_2_T_x_), metal oxides, organic dye degradation, advanced functional materials

## Abstract

As the demand for clean water continues to rise, the development of reliable and environmentally sustainable purification methods has become increasingly important. In this study, we describe the production and characterization of electrospun polyimide (PID) nanofibers modified with MXene (Ti_3_C_2_Tx), tungsten trioxide (WO_3_), and titanium dioxide (TiO_2_) nanomaterials for improved photocatalytic degradation of rhodamine 6G (R6G), a model organic dye. Superior photocatalytic performance was achieved by suppressing electron–hole recombination, promoting efficient charge carrier separation, and the significant increase in light absorption through the addition of metal oxide nanowires and MXene to the PID matrix. Comprehensive characterization confirms a core–shell nanofiber architecture with TiO_2_, WO_3_, and MXene effectively integrated and electronically coupled, consistent with the observed photocatalytic response. The PID/TiO_2_/WO_3_/MXene composite exhibited the highest photocatalytic activity among the tested configurations, degrading R6G by 74% in 90 min of light exposure. This enhancement was ascribed to the synergistic interactions between MXene and the metal oxides, which reduced recombination losses and promoted effective charge transfer. The study confirms the suitability of PID-based hybrid nanofibers for wastewater treatment applications. It also points toward future directions focused on scalable production and deployment in the field of environmental remediation.

## 1. Introduction

Water scarcity is becoming increasingly challenging and difficult to manage in many regions worldwide. This is due in part to rapid population growth, expanding industrial development, and the limited adaptability of conventional purification technologies [1]. Textile manufacturing is a significant source of water pollution, releasing large volumes of wastewater containing synthetic dyes. These compounds tend to be chemically stable, environmentally persistent, and harmful to aquatic ecosystems [2]. The challenges of operational complexity, high costs, and the production of secondary waste remain significant and critical [3]. These challenges have encouraged researchers to explore new treatment technologies that offer greater reliability, can be scaled up more easily, and have a lower environmental impact. Among them, photocatalysis has gained recognition as a promising approach for breaking down organic pollutants in water through a light-driven, energy-efficient process. Semiconductor photocatalysts such as TiO_2_, ZnO, WO_3_, CdS, and ZnS are widely studied for dye and pollutant removal, with recent work emphasizing defect engineering and heterostructure design to enhance activity under illumination [4,5]. These materials are favored for their chemical robustness, environmental compatibility, and relatively low production costs [6]. Nevertheless, their practical use remains limited by several intrinsic drawbacks. These include insufficient absorption in the visible range, high rates of electron–hole recombination, and considerable band gap energies, all of which reduce overall photocatalytic efficiency [7,8].

Improving photocatalytic materials often comes down to two main ideas. The initial step in the process is to reduce the particle size, thereby exposing a larger surface area, which helps increase the number of active sites [9,10]. The second approach involves the combination of different semiconductors into heterostructures, which facilitate the movement of charges more freely and maintain their separation for more extended periods. This improves how light is absorbed and utilized, and reduces the likelihood of recombination [11,12]. These changes are most effective when the material structure can be easily adjusted, especially at the nanoscale, where minor differences have significant effects.

Among the various fabrication methods, electrospinning stands out due to its ability to easily produce long, high-porosity fibers with a large surface area. This method is compatible with multiple materials, including polymers and ceramic additives, and allows control over fiber structure, spacing, and surface properties [13]. The fibrous networks created in this manner are beneficial for photocatalysis, as they provide light and reactants with better access to active regions and can be modified to accommodate various reactions.

The incorporation of conductive nanomaterials into photocatalytic systems has recently shown great potential in enhancing charge transport and prolonging the lifetime of photogenerated carriers. MXenes, a family of two-dimensional transition metal carbides and carbonitrides first introduced by Gogotsi and co-workers in 2011 [14], have attracted particular attention. Ti_3_C_2_T_x_, the most extensively studied MXene, exhibits excellent electronic conductivity and strong hydrophilicity, along with surface terminations (-OH, -O, -F) that enable tunable interfacial interactions. In hybrid systems, MXenes can function as efficient electron sinks, facilitating charge separation and improving light absorption. For instance, Zheng et al. [15] demonstrated that incorporating Ti_3_C_2_T_x_ into a TiO_2_ matrix led to enhanced dye degradation performance under light irradiation due to improved charge separation.

In this study, we report a composite nanofiber system based on electrospun polyimide (PID) incorporating TiO_2_ and WO_3_ nanowires along with Ti_3_C_2_T_x_ MXene sheets. This design aims to exploit the synergistic effects of the metal oxides and MXene within a well-structured fibrous network to enhance the photocatalytic degradation of rhodamine 6G (R6G), a model organic dye. AFM, SEM, TEM, XPS, UV-Vis spectroscopy, photoluminescence analysis, and photocatalytic testing under simulated solar irradiation were used to characterize the resulting materials. Our findings indicate that incorporating MXene and metal oxides into the electrospun PID matrix significantly enhances photocatalytic performance, driven by improved light absorption, efficient interfacial charge transfer, and suppressed charge recombination. These results underscore the promise of PID-based composite fibers for next-generation water purification technologies.

## 2. Materials and Methods

### 2.1. Reagents and Chemicals

Polyimide pellets (P8TM SG) (PID) were purchased from HP Polymer Inc. (Lenzing, Austria). N,N-Dimethylacetamide (99.8%) (DMA) was obtained from PENTA Chemicals (Prague, Czech Republic). Titanium dioxide (TiO_2_) and tungsten trioxide (WO_3_) nanowires, along with titanium aluminum carbide (Ti_3_AlC_2_, MAX phase), were sourced from Sigma-Aldrich (St. Louis, MO, USA). Unless specified, reagents were used as received without further purification; solutions were prepared fresh in deionized water (18.2 megaohm centimeter). Dispersions were sonicated to homogenize and, when required, passed through a 0.22 micrometer syringe filter.

### 2.2. Synthesis of MXenes

Ti_3_C_2_T_x_ MXene was prepared by etching Ti_3_AlC_2_ (MAX-phase) using the MILD method in a solution of lithium fluoride (LiF) in hydrochloric (HCl) acid. The etching mixture was prepared from 40 mL of 12 M HCl (37%) and 10 mL of DI water, and then 3.2 g of LiF was dissolved in this solution. The mixture was placed in a 50 mL plastic container. Next, 2 g of Ti_3_AlC_2_ powder with particle sizes less than 40 μm was gradually added to the etching solution under continuous stirring for 24 h at 25 °C. The MXene slurry was then rinsed with DI water via repetitive centrifugation. As-prepared MXene slurry is further processed to obtain a colloidal solution of separated MXene flakes using a mild delamination procedure.

### 2.3. Solutions Preparation and Electrospinning

**Figure 1:**
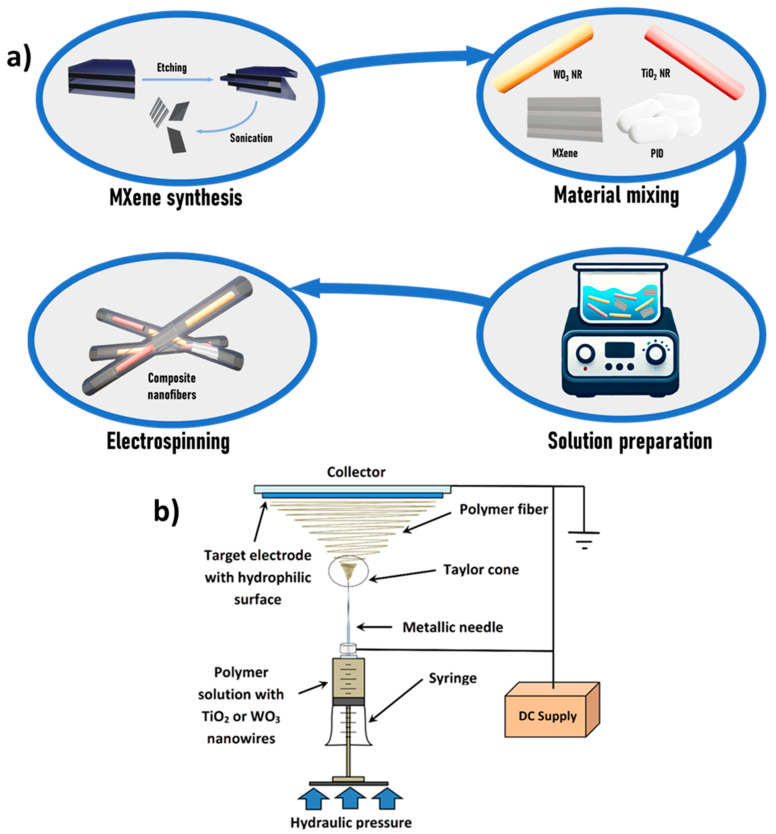
(**a**) Schematic representation of the composite nanofiber synthesis process. (**b**) Experimental setup used for nanofiber fabrication.

The schematic representation of the solution preparation and electrospinning processes is shown in Figure 1a. The polymeric solution for electrospinning was prepared by dissolving 16% *w*/*w* polyimide (PID) pellets in N,N-dimethylacetamide (DMA). The mixture was stirred at 250 rpm for 24 h at room temperature to ensure complete dissolution.

Solutions containing additives were prepared following the same procedure, with an additional 5% *w*/*w* of each planned additive mixed with the 16% *w*/*w* PID pellets. The following composite solutions were prepared: pristine PID, PID/TiO_2_, PID/TiO_2_/MXene, PID/WO_3_, PID/WO_3_/MXene, and PID/TiO_2_/WO_3_/MXene.

A custom-made electrospinning setup was used for fiber fabrication (Figure 1b). The prepared solutions were loaded into 5 mL plastic syringes. Standard sterile injection needles (0.60 mm in diameter, 40 mm length) were modified by cutting the tips to ensure a symmetric electric field and were subsequently used for electrospinning. An antistatic polypropylene spunbond non-woven mat (surface density 18 g/m^2^) was attached to the grounded conveyor belt collector, maintaining a 200 mm distance between the needle tip and the collector. An electric field of 10 kV was applied during electrospinning, and the solution was dispensed at a constant flow rate of 5 mL/hour using a syringe pump.

### 2.4. Characterization

The structural properties of the produced nanofibers were investigated by scanning electron microscopy (SEM) (JEOL JSM-7001F), equipped with an energy-dispersive X-ray (EDX) analyzer with an accelerating voltage of 5–10 kV and working distance of 8–12 mm, and high-resolution transmission electron microscopy (HR-TEM) (JEOL ARM 200F, 200 kV) (JEOL Ltd., Akishima, Tokyo, Japan), also equipped with an EDX analyzer. AFM Bruker ICON was employed to study properties of MXene flakes (Bruker Corporation, Billerica, MA, USA). X-ray photoelectron spectroscopy (XPS) was performed using a KRATOS Axis DLD spectrometer with monochromatic Al Kα (1486.6 eV) (Kratos Analytical Ltd., Manchester, UK); survey and core levels were at 160 eV and 20 eV pass energies (energy resolution ~0.4 eV); spectra was referenced to C 1s at 284.8 eV and fitted using a Shirley background and mixed Gaussian–Lorentzian profiles. We used Renishaw in-Via micro-Raman system with plane-polarized lasers at λexc = 633 nm with a 50× objective, a laser power of ≤1 mW, and an over measurement range with two to four accumulations and calibrated to the 520.7 cm^−1^ Si line. FTIR spectra by JASCO FT/IR-4700 (with ATR PRO ONE) (JASCO Corporation, Hachioji, Tokyo, Japan) were collected in ATR mode over 4000–400 cm^−1^ at 4 cm^−1^ resolution, and the spectra were baseline-corrected and normalized before analysis. The optical properties, including absorbance and photoluminescence, were examined using an Ocean Optics QE65 Pro spectrophotometer (Ocean Optics, Inc., Dunedin, FL, USA). Diffuse reflectance measurements were performed using an Ocean Optics QE Pro spectrometer, coupled with an integrating sphere, with subsequent conversion via the Kubelka–Munk function F(R), and band gaps were extracted from Tauc plots with n = 2 by linear extrapolation. However, no significant changes or additional insights were observed compared with other characterization methods.

### 2.5. Photodegradation Tests

Photocatalytic degradation tests were performed using a 300 W xenon (Xe) lamp as the irradiation, with Rhodamine 6 G (R6G) selected as the model organic dye. The R6G solution (pH ~7.0) was prepared at a concentration of 10^−2^ mg/mL. Photodegradation used a 1 × 2 cm nanofiber coupon (2.0 cm^2^) mounted on a plastic holder; although mats differed between samples, photocatalyst loading was controlled by applying an identical aliquot of the same dispersion per coupon (single-sided) under identical drying/curing conditions, and each test used 2.0 mL of Rhodamine 6G solution. UV-Vis light irradiation of the samples was conducted in a quartz 3.5 mL cuvette positioned 10 cm from the light source. The absorption spectrum was continuously recorded using an Ocean Optics USB spectrometer (QE65-Pro) (Ocean Optics, Inc., Dunedin, FL, USA). The remaining concentration of R6G in the solution was determined by analyzing the absorbance spectrum at λ = 530 nm. A calibration curve of absorbance was used to evaluate the photocatalytic activity of the samples. 

## 3. Results and Discussion

### 3.1. Structure Characterization

**Figure 2:**
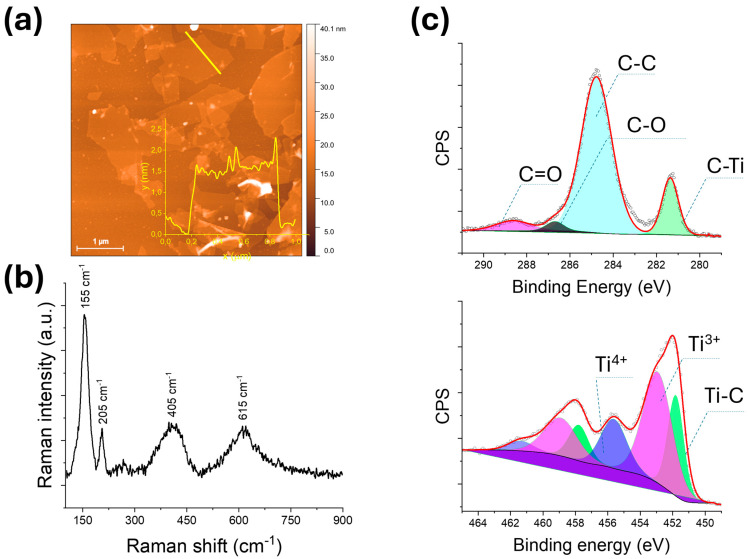
(**a**) AFM image and height profile of an exfoliated Ti_3_C_2_T_x_ MXene flake. (**b**) Raman spectrum of Ti_3_C_2_T_x_. (**c**) High-resolution XPS spectra of the C 1s and Ti 2p regions.

Prior to their incorporation into polymeric nanofibers, the morphological characteristics of the as-prepared MXene flakes were analyzed using AFM. Figure 2a shows MXene flakes deposited on a flat substrate. The flake displays a uniform and planar morphology with sharply defined edges, indicating successful exfoliation of the Ti_3_C_2_T_x_ MXene. The average lateral size of the flake is approximately 1.5 μm, consistent with micrometer-scale flakes typically obtained through mild delamination techniques. The inset in Figure 2a shows the height profile along a selected cross-section of the flake, with an average thickness of approximately 1.5 nm. This thickness corresponds to single- to few-layer Ti_3_C_2_T_x_ MXene sheets as reported in previous studies [16].

The structural features of the exfoliated Ti_3_C_2_T_x_ MXene were first examined by Raman spectroscopy using a 633 nm excitation laser (Figure 2b). The spectrum displays broad and distinct peaks characteristic of few-layer MXene with surface terminations and defect-induced disorder. The prominent peak at 155 cm^−1^ corresponds to the A_1g_ mode of out-of-plane Ti vibrations [17], confirming the preservation of the Ti–C network. A secondary peak at 205 cm^−1^ is attributed to the Eg mode, related to in-plane vibrations. Additionally, bands observed at 405 cm^−1^ and 615 cm^−1^ are associated with Ti–O and Ti–OH surface terminations [16], respectively, and possible lattice distortions caused by delamination or oxidation [18].

XPS analysis (Figure 2c) provided further insight into the surface chemistry of Ti_3_C_2_T_x_. The C 1s spectrum revealed peaks at 281.3 eV (C–Ti), 284.7 eV (C–C/C–H), 286.7 eV (C–O/C–OH), and 288.6 eV (C=O/O–C=O) [19], confirming the coexistence of carbide and oxidized carbon species. The Ti 2p spectrum showed three distinct components: Ti–C (451.8 eV), Ti^3+^ (~457.7 eV), and Ti^4+^ (~458.9 eV), indicating partial surface oxidation, likely forming a thin TiO_2_ shell while preserving the carbide-rich core. These analyses confirm the successful synthesis of MXene with maintained structural integrity.

The as-synthesized polymeric nanofibers incorporating MXene, TiO_2_, TiO_2_/MXene, WO_3_, WO_3_/MXene, and TiO_2_/WO_3_/MXene were thoroughly examined using SEM coupled with EDX analysis, as well as TEM, to gain a comprehensive understanding of the composites’ morphology and internal structure.

**Figure 3:**
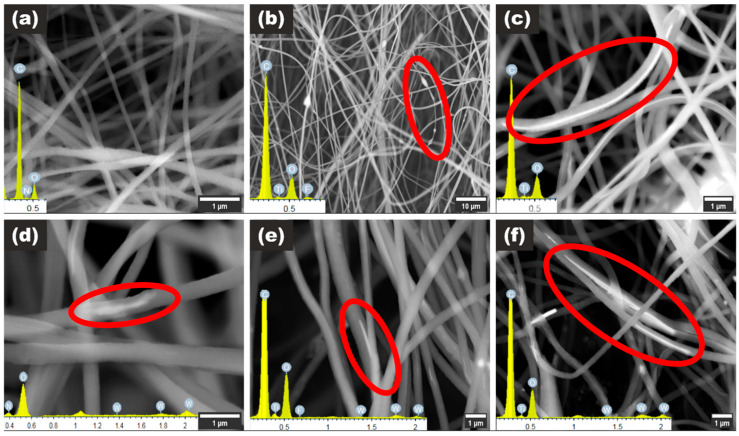
(**a**) SEM images of PID, (**b**) PID/MXene, (**c**) PID/TiO_2_, (**d**) PID/WO_3_, (**e**) PID/WO_3_/MXene, and (**f**) PID/TiO_2_/WO_3_/MXene nanofibers. Inset graphs represent corresponding EDX measurements. Red circles highlight areas within the nanofiber mats where nanoparticles are incorporated into the fibers.

In Figure 3a, the morphology of the pristine polymeric nanofibers is displayed, with EDX analysis confirming characteristic polymeric peaks. The morphology of the PID/MXene composite, shown in Figure 3b, maintains a nanofiber structure but exhibits the formation of polymeric beads. The occurrence of these beads is attributed to changes in net charge density due to the introduction of MXene nanoflakes [20]. The EDX pattern in Figure 3b confirms the presence of Ti_3_C_2_ MXenes, as indicated by the clear Ti and F peaks. The fluorine peak is associated with surface termination groups remaining on the Ti_3_C_2_ surface after etching the Ti_3_AlC_2_ MAX phase [21].

The most uniform nanofibers, in terms of additive dispersion, are those formed from PID combined with commercial TiO_2_ nanowires. As shown in Figure 3c, the TiO_2_ nanowires embedded within the PID nanofibers form a coaxial (core–shell) structure, which is analogous to the insulation surrounding traditional wires. This structure enables the fabrication of flexible, conductive, and insulated nanoscale wires suitable for advanced electronic applications.

Figure 3d–f illustrates the morphologies of PID/WO_3_, PID/WO_3_/MXene, and PID/TiO_2_/WO_3_/MXene composites. These fiber mats display an uneven distribution of WO_3_, TiO_2_, and MXene within the fibers. This non-uniformity could result from electrostatic interactions among the components, sedimentation, or blockages in the electrospinning needle [22]. However, the composite containing TiO_2_ nanowires (Figure 3f) demonstrates improved uniformity and distribution of solid materials within the nanofiber core.

**Figure 4:**
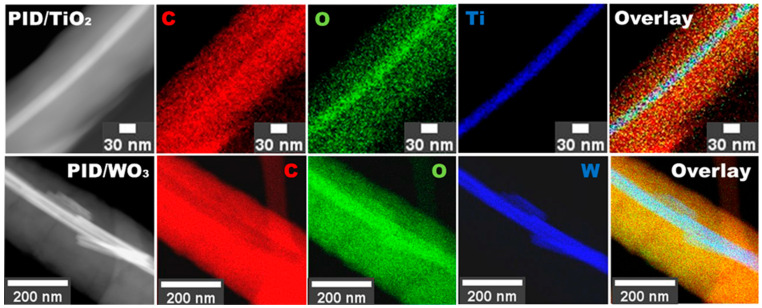
TEM images of PID/TiO_2_ (upper line), PID/WO_3_ (lower line).

TEM analysis was conducted to investigate further the structural features of PID nanofibers incorporating TiO_2_ and WO_3_, as shown in Figure 4. The results reveal the formation of a TiO_2_-core/PID-shell structure (Figure 4, upper row), where the TiO_2_ nanowire is centrally embedded within the PID nanofiber (the image labeled “Ti” represents elemental titanium). In contrast, the PID/WO_3_ nanofibers exhibit an irregular distribution of WO_3_ nanowires or their fragments. The presence of WO_3_ is confirmed by the elemental mapping of tungsten (labeled “W”) in the lower row of Figure 4.

### 3.2. Chemical and Optical Properties of Produced Nanocaomposites

To investigate the chemical structure and composition of the developed nanocomposite system, a combination of Raman spectroscopy, XPS, FTIR, UV-Vis, and PL analyses was employed.

**Figure 5:**
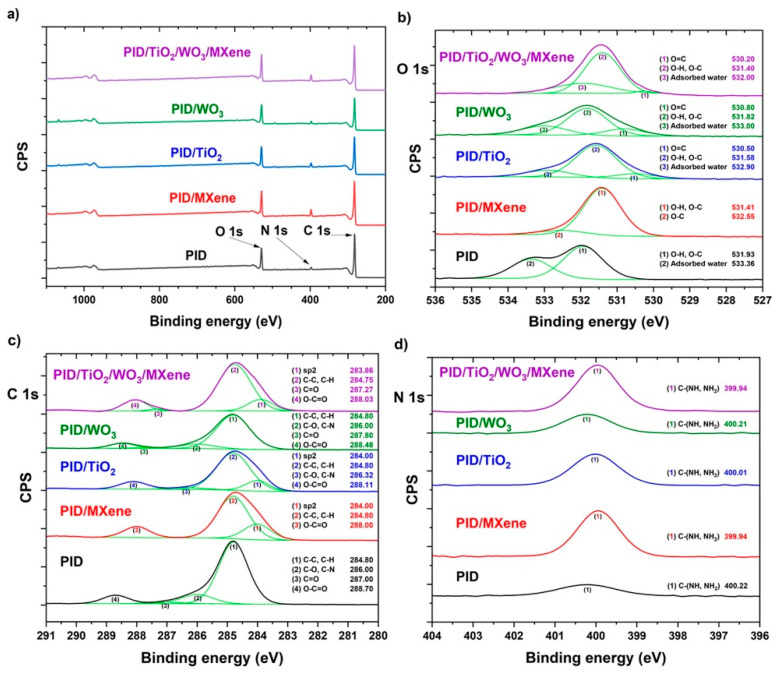
(**a**) Full-range XPS spectra, and (**b**) high-resolution O 1s, (**c**) C 1s, (**d**) N 1s spectra of PID-based nanofibers. Corresponding binding energy values obtained by the deconvolution of the detected peaks are shown in the insets.

To evaluate the impact of MXene and metal oxide incorporation into the polyimide (PID) matrix, high-resolution XPS was also conducted on the electrospun nanofibers (Figure 5). In the O 1s region (Figure 5b), the dominant peak at ~530 eV corresponds to O=C bonds, while those at ~531–532 eV are attributed to O–H and O–C functionalities [23]. The appearance of a minor peak near ~533 eV, associated with adsorbed water, suggests slight non-uniformity in the electrospun mats [23]. A noticeable increase in oxygen content and subtle shifts in peak positions were observed upon addition of MXene or TiO_2_/WO_3_/MXene, suggesting interactions between the polymer and the surface terminations of the nanomaterials [24].

The C 1s spectrum (Figure 5c) exhibited consistent peaks at ~283 eV (sp^2^ C) [25], ~284.8 eV (C–C/C–H), and ~288 eV (O–C=O), with no significant changes following admixture incorporation [26]. However, the ~286 eV peak (C–O, C–N) showed reduced intensity, indicating possible modification of the polyimide backbone or matrix–additive interactions. The weak and unstable C=O peak at ~287 eV was only occasionally detected, suggesting no significant structural disruption [26].

Nitrogen bonding was assessed via the N 1s spectrum (Figure 5d), where the peak at ~400 eV, assigned to C–(NH, NH_2_) groups, increased in intensity upon additive incorporation [26]. This enhancement suggests stronger nitrogen interactions between the polymer and embedded nanomaterials, particularly MXene.

Complementary FTIR analysis (Figure S1) confirmed the preservation of the polyimide molecular structure across all nanocomposite samples. The presence of characteristic absorption bands, including C=O stretching (~1720 cm^−1^), C–N stretching (~1375 cm^−1^), and imide ring deformation (~725 cm^−1^), was consistently observed [27]. The absence of new peaks or significant shifts indicates that TiO_2_, WO_3_, and MXene were physically embedded, rather than chemically altering the polyimide matrix.

**Figure 6:**
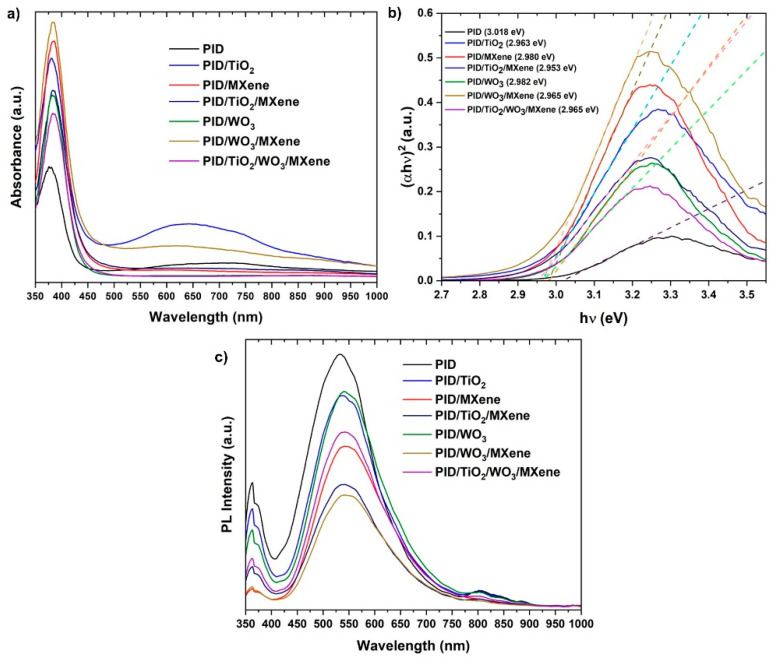
(**a**) UV-Vis absorption spectroscopy, (**b**) Tauc plot, and (**c**) photoluminescence spectra of different samples.

The optical properties of the nanofibers were evaluated by UV-vis spectroscopy (Figure 6a). The pristine PID exhibited strong UV absorption and limited visible activity [28]. The incorporation of TiO_2_ and WO_3_ enhanced UV absorption [29], while the addition of MXene significantly broadened the absorption into the visible region due to its metallic character and dark coloration [30]. The Tauc plot-derived [31] band gaps (Figure 6b) revealed a slight reduction (~50 meV) in Eg for the composite samples, indicative of enhanced electronic interaction and interfacial charge transfer.

PL spectra (Figure 6c) revealed a broad emission peak centered at approximately 550 nm [32] under 360 nm excitation [33], characteristic of PID. A substantial reduction in PL intensity was observed across all composite samples, with the most pronounced quenching occurring in those containing MXene. This decrease in emissions suggests enhanced charge carrier separation and reduced radiative recombination, likely resulting from improved electrical conductivity and more effective interfacial charge transfer within the hybrid nanofiber network.

These results confirm that the incorporation of MXene and metal oxides into PID nanofibers leads to enhanced surface chemistry, broadened optical absorption, and improved charge carrier dynamics, all of which are beneficial for photocatalytic applications.

Although Figure 6b gives only the optical gaps, the band edges of the oxide phases are well documented. In anatase TiO_2_, the conduction-band minimum is placed at about −0.24 V vs. NHE by electronegativity arguments [34], and flat-band measurements in aqueous media cluster near −0.16 V [35]; with a band gap of ~3.2 eV, this sets the valence band at ~+2.96 V vs. NHE. For pristine WO_3_, the conduction-band edge is reported close to +0.44 V vs. RHE [36], which, together with its gap, locates the valence band at about +3.0–3.1 V vs. RHE. In such an alignment, photoexcited electrons in TiO_2_ have a downhill route into the MXene (Ti_3_C_2_T_x_) conductor, while holes reside on the deep WO_3_ valence band at potentials sufficient for oxidative steps. Comparable band-alignment analyses reach the same band-alignment scenario [37].

### 3.3. Dye Photodegradation

The photocatalytic activity of the synthesized PID nanofiber composites was evaluated through the degradation of R6G under Xe lamp irradiation. UV-Vis absorption spectra of the R6G solution were recorded at various time intervals to monitor the degradation process. The characteristic absorption peak of R6G at approximately 530 nm gradually decreased with increasing irradiation time, indicating effective photodegradation facilitated by the nanofiber composites.

**Figure 7:**
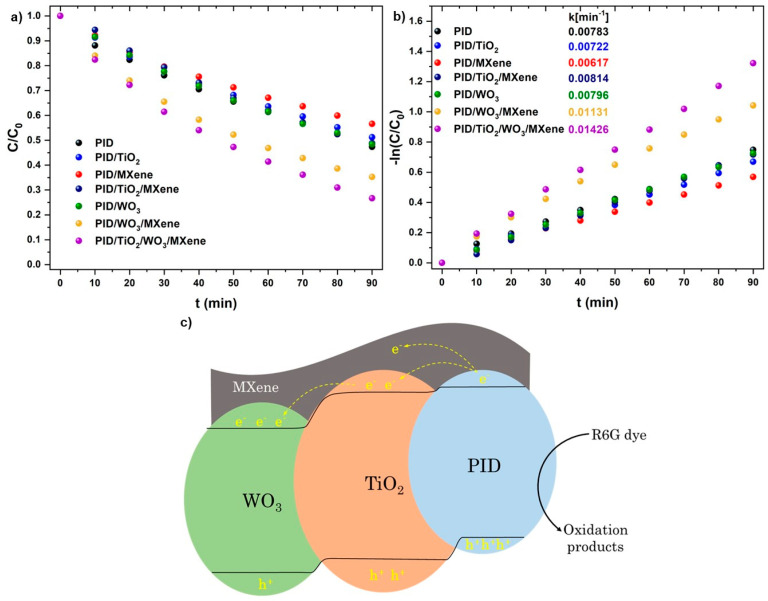
(**a**) R6G decolorization and (**b**) reaction kinetics over pristine and various hybrid PID nanofibers; (**c**) the mechanism of charge transfer in heterojunction during the photodegradation process on PID/TiO_2_/WO_3_/MXene nanofiber.

Figure 7a presents a comparison of photodegradation efficiencies among the investigated samples: PID, PID/MXene, PID/TiO_2_, PID/TiO_2_/MXene, PID/WO_3_, PID/WO_3_/MXene, and PID/TiO_2_/WO_3_/MXene. After 90 min of light exposure, the degradation efficiencies were calculated to be 53%, 43%, 49%, 51.5%, 51.5%, 65%, and 74%, respectively. The PID/TiO_2_/WO_3_/MXene composite exhibited the highest degradation efficiency, clearly demonstrating the synergistic enhancement resulting from the integration of TiO_2_, WO_3_, and MXene.

To gain deeper insight into the reaction mechanism, the photodegradation kinetics were evaluated using a pseudo-first-order model, expressed as

ln(C_0_/C) = k × t, (1)

where k is the apparent rate constant (min^−1^), t is the irradiation time (min), and C_0_ and C are the initial and time-dependent dye concentrations, respectively. The calculated k values are shown in Figure 7b. The highest rate constant, 0.01426 min^−1^, was observed for the PID/TiO_2_/WO_3_/MXene composite, which is approximately 1.82 times greater than that of the pristine PID sample (0.00783 min^−1^), confirming its superior photocatalytic performance. To benchmark the efficiency of the fabricated PID-based nanofibers against other photocatalytic systems reported in the literature, we compared their kinetic parameters and overall degradation efficiencies under different experimental conditions. A summary of representative photocatalysts, pollutants, irradiation sources, and corresponding removal efficiencies is provided in Table 1.

The remarkable enhancement in photocatalytic activity can be attributed to improved charge separation and interfacial charge transfer between TiO_2_, WO_3_, MXene, and the polyimide matrix. This finding is consistent with previous reports on polyimide-based hybrid photocatalysts [46]. The degradation of R6G is primarily driven by photogenerated holes (h^+^), which directly oxidize dye molecules. Hydroxyl radicals (•OH) act as secondary reactive species, further contributing to the breakdown of R6G into less harmful intermediates. The formation of these species is attributable to the oxidation of water or hydroxide ions by excess photogenerated holes.

Based on the aforementioned conduction and valence band positions of WO_3_, superoxide formation is unlikely, and photogenerated holes are expected to be the main reactive species [36]. Studies on WO_3_ composites further show that quenching holes causes the largest drop in photocatalytic activity [47].

TiO_2_ and WO_3_ function as complementary photocatalysts. TiO_2_ facilitates efficient electron transfer due to its conduction-band position, while WO_3_ stabilizes photogenerated holes through its deeper valence band. MXene, serving as a highly conductive co-catalyst, enhances electron mobility and suppresses charge recombination. The combination of these components yields a multi-component photocatalytic system that harnesses solar energy more effectively, leading to enhanced degradation efficiency. Additionally, in our hybrid, the Ti_3_C_2_T_x_ layer acts as an electron sink that draws electrons from the oxides and improves charge separation, leaving the oxidative step to the holes on the oxide domains rather than electrons [48].

We evaluated reusability in five consecutive runs on the same sample (Figure S2). After each run, the coupon was removed, rinsed with deionized water, dried under a gentle argon flow, and then returned to fresh dye solution. In the first run, the removal was about 72%, which corresponds to a concentration ratio of C over C_0_ of roughly 0.28. The second and third runs stayed close to 70%. A slight decline appeared thereafter, and by the fifth run, the removal was about 66% with a concentration ratio near 0.34. This modest loss is consistent with some dye remaining on the surface and partial blocking of active sites, and overall, the results indicate stable performance upon reuse.

## 4. Conclusions

In this study, electrospun polyimide (PID) nanofibers incorporating TiO_2_, WO_3_, and MXene (Ti_3_C_2_T_x_) nanomaterials were successfully fabricated and systematically evaluated for their photocatalytic efficiency in the degradation of rhodamine 6G (R6G). The integration of metal oxide nanowires and MXene into the PID matrix significantly enhanced photocatalytic activity by improving light absorption, promoting effective charge carrier separation, and suppressing electron–hole recombination. A comprehensive structural and chemical characterization using SEM, TEM, XPS, UV-Vis spectroscopy, and photoluminescence analyses confirmed the successful incorporation of the nanomaterials and the formation of well-organized core and shell fiber architectures. The PID/TiO_2_/WO_3_/MXene composite exhibited the best photocatalytic performance among the samples, achieving 74% dye degradation within 90 min. This improvement likely results from the combined effects of the three constituents, which improved charge mobility across interfaces and suppressed charge carrier recombination. Core–shell nanofiber platform integrating TiO_2_, WO_3_, and MXene in a polyimide scaffold outperforms the PID, PID/TiO_2_, and PID/WO_3_ controls in Rhodamine 6G removal at neutral pH. The TiO_2_/WO_3_/MXene coupon removes about seventy-four percent in ninety and maintains activity over five reuse cycles, consistent with a hole-driven pathway supported by the band positions and optical data. These results highlight the potential of PID-based hybrid nanofibers as advanced materials for sustainable wastewater treatment. Future work should prioritize scaling up the synthesis process, assessing the long-term stability and recyclability of the composites, and extending their application to a broader range of organic pollutants under varying environmental conditions.

## Figures and Tables

**Table 1:** Comparison of photodegradation performance of various photocatalysts.

Photocatalyst	Pollutant (C_0_, mg/L)	Lamp, Power (W)	Spectrum	Time (min)	k (min^−1^)	Removal (%)	Ref.
PID/TiO_2_/WO_3_/MXene	R6G (10)	Xenon, 300	UV–Vis	90	0.01426	74	this work
AgNPs/TiO_2_/Ti_3_C_2_T_x_	MB (10)	Mercury, 400	UV	30	0.162	99	[38]
	RhB (10)	Mercury, 400	UV	40	0.143	99	
	MB (10)	Solar simulator	Sunlight	120	0.028	96	
	RhB (10)	Solar simulator	Sunlight	120	0.020	88	
Ag_3_PO_4_/TiO_2_@Ti_3_C_2_ (Petals)	MB (10)	Xenon, 300	Visible	40	0.289	94–100	[39]
Ti_3_C_2_@TiO_2_/g-C_3_N_4_ (ternary)	RhB (10)	Xenon, 300	Visible	120	0.01575	-	[40]
g-C_3_N_4_	RhB (10)	Xenon, 300	Visible	120	0.01575	-	[40]
TiO_2_(B)/Ti_3_C_2_/Ag_3_PO_4_	RhB (10)	Visible lamp	Visible	60	0.182–0.345	90–100	[41]
TiO_2_(B)	RhB (10)	Visible lamp	Visible	60	0.008	-	[41]
TiO_2_(B)/20% Ti_3_C_2_	RhB (10)	Visible lamp	Visible	60	0.011	-	[41]
MXene/g-C_3_N_4_ (1 wt% Ti_3_C_2_)	MB (10)	Halogen, 500	Visible	180	~0.0051	~60	[42,43]
TiO_2_@Ti_3_C_2_ (baseline in Petals work)	RhB (9)	Solar simulator	Sunlight	20	0.0093	-	[39]
Supported TiO_2_ (Raschig rings)	R6G (5)	White light	Visible	-	0.025	66–77	[44]
Pristine Ti_3_C_2_	Congo Red (10)	Visible lamp	Visible	120	-	~12	[45]

## Data Availability

The original contributions presented in this study are included in the article/Supplementary Materials. Further inquiries can be directed to the corresponding author.

## Supplementary Materials

The following supporting information can be downloaded at https://www.mdpi.com/article/10.3390/nano15171371/s1, Figure S1: FTIR spectra of the electrospun nanofibers. Figure S2. Recyclability of the photodegradation for the PID/TiO_2_/WO_3_/MXene sample over five consecutive runs (90 min each).

## Abbreviations

The following abbreviations are used in this manuscript:

PID	Polyimide
R6G	Rhodamine 6G
SEM	Scanning electron microscopy
TEM	Transmission electron microscopy
XPS	X-ray photoelectron spectroscopy
PL	Photoluminescence
EDX	Energy-dispersive X-ray spectroscopy
HR-TEM	High-resolution transmission electron microscopy
FTIR	Fourier-transform infrared spectroscopy
AFM	Atomic force microscopy
