# Supplementary material for: Electrospun Polyimide Nanofibers Modified with Metal Oxide Nanowires and MXene for Photocatalytic Water Purification
Source: Nanomaterials (Basel). 2025 Sep 5;15(17):1371. doi: 10.3390/nano15171371 (PMC12430064; doi:10.3390/nano15171371)
Supplement: Supplementary file 1 [file nanomaterials-15-01371-s001.zip › nanomaterials-3805506-supplementary.pdf]

Supporting Information

Electrospun Polyimide Nanofibers Modified with Metal Oxide Nanowires and MXene for Photocatalytic Water Purification

Andrii Lys [1], Valerii Myndrul [2], Mykola Pavlenko [1], Błażej Anastaziak [1], Pavel Holec [3], Kateřina Vodseďálková [4], Emerson Coy [1], Mikhael Bechelany [5] and Igor Iatsunskyi [1,*]

1 NanoBioMedical Centre, Adam Mickiewicz University, Poznan, Poland; andrii.lys@amu.edu.pl (A.L.); mykpav@amu.edu.pl (M.P.); blazej.anastaziak@amu.edu.pl (B.A.) coyeme@amu.edu.pl (E.C.);
2 Sensor Engineering Department, Faculty of Science and Engineering, Maastricht University, Maastricht, the Netherlands; valerii.myndrul@maastrichtuniversity.nl
3 Department of Nonwovens and Nanofibrous Materials, Faculty of Textile Engineering, Technical University of Liberec, Liberec, Czech Republic; pavel.holec@tul.cz
4 Nanopharma a. s, Pardubice, Czech Republic; vodsedalkova@nanopharma.cz
5 European Institut of Membranes (IEM) – UMR 5635, University of Montpellier, ENSCM, CNRS, Montpellier, France; Mikhael.bechelany@umontpellier.fr
* Correspondence: igoyat@amu.edu.pl;

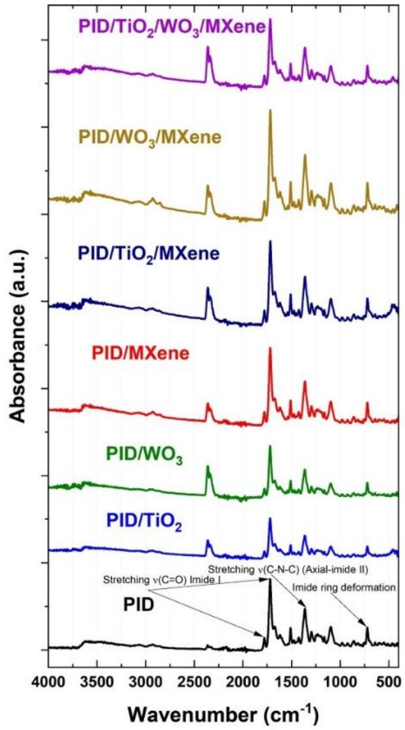

Figure S1. FTIR spectra of the electrospun nanofibers.

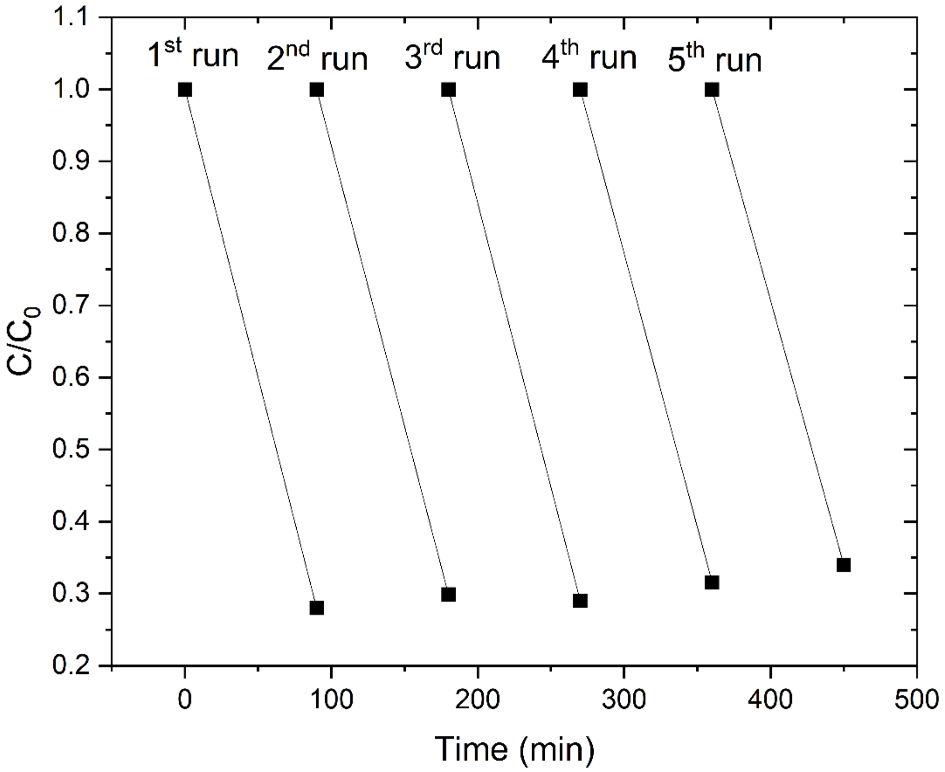

Figure S2. Recyclability of the photodegradation for the PID/TiO_2/WO_3/MXene sample over five consecutive runs (90 min each).
